# Steatomatous Cyst upon the Tongue

**Published:** 1863-05

**Authors:** 


Steatomatous Cyst under the Tongue.—Prof. Alfred C. Post presented a cyst and its contents, taken from beneath the mucous membrane under the tongue. It is interesting on account of the situation from which it was taken. He at first supposed it to be a ranula, but found it to be an encysted tumor filled with steatomatous matter, similar to that filling cysts found under the skin, as proved by critical examination made by Dr. H. B. Sands. He is not aware of a cyst of this kind having been found in this situation before.

*From the New York Pathological Society.*

